# The osteocytic actions of glucocorticoids on bone mass, mechanical properties, or perilacunar remodeling outcomes are not rescued by PTH(1-34)

**DOI:** 10.3389/fendo.2024.1342938

**Published:** 2024-07-18

**Authors:** Cristal S. Yee, Christoforos Meliadis, Serra Kaya, Wenhan Chang, Tamara Alliston

**Affiliations:** ^1^ Department of Orthopaedic Surgery, University of California, San Francisco, San Francisco, CA, United States; ^2^ Endocrine Research Unit, San Francisco Veterans Affairs Medical Center, University of California, San Francisco, CA, United States

**Keywords:** osteocyte, glucocorticoids, prednisolone, parathyroid hormone (PTH), PTH (1-34), perilacunar canalicular remodeling, osteocytic osteolysis, bone

## Abstract

Glucocorticoids (GC) and parathyroid hormone (PTH) are widely used therapeutic endocrine hormones where their effects on bone and joint arise from actions on multiple skeletal cell types. In osteocytes, GC and PTH exert opposing effects on perilacunar canalicular remodeling (PLR). Suppressed PLR can impair bone quality and joint homeostasis, including in GC-induced osteonecrosis. However, combined effects of GC and PTH on PLR are unknown. Given the untapped potential to target osteocytes to improve skeletal health, this study sought to test the feasibility of therapeutically mitigating PLR suppression. Focusing on subchondral bone and joint homeostasis, we hypothesize that PTH(1-34), a PLR agonist, could rescue GC-suppressed PLR. The skeletal effects of GC and PTH(1-34), alone or combined, were examined in male and female mice by micro-computed tomography, mechanical testing, histology, and gene expression analysis. For each outcome, females were more responsive to GC and PTH(1-34) than males. GC and PTH(1-34) exerted regional differences, with GC increasing trabecular bone volume but reducing cortical bone thickness, stiffness, and ultimate force. Despite PTH(1-34)’s anabolic effects on trabecular bone, it did not rescue GC’s catabolic effects on cortical bone. Likewise, cartilage integrity and subchondral bone apoptosis, tartrate-resistant acid phosphatase (TRAP) activity, and osteocyte lacunocanalicular networks showed no evidence that PTH(1-34) could offset GC-dependent effects. Rather, GC and PTH(1-34) each increased cortical bone gene expression implicated in bone resorption by osteoclasts and osteocytes, including *Acp5, Mmp13, Atp6v0d2, Ctsk*, differences maintained when GC and PTH(1-34) were combined. Since PTH(1-34) is insufficient to rescue GC’s effects on young female mouse bone, future studies are needed to determine if osteocyte PLR suppression, due to GC, aging, or other factors, can be offset by a PLR agonist.

## 1 Introduction

Common clinical therapies for immune suppression or osteoporosis include glucocorticoids and parathyroid hormone-based therapies, respectively. Therefore, understanding the effects of these common clinical therapies on skeletal health is important. Though the effects of these therapies alone or in combination on several aspects of bone health have been extensively studied in humans (1–3) and rodents (4–11), their combined effect on osteocyte-mediated perilacunar resorption, which is a target of both therapies independently, remains unclear.

Osteocytes are embedded in the bone matrix within the lacunar canalicular network (LCN). Osteocyte dendrites extend through canaliculi to communicate with other cells to regulate bone homeostasis, among other osteocytic functions. The LCN and bone quality are actively maintained by osteocytes through the homeostatic process of perilacunar canalicular remodeling (PLR), in which osteocytes resorb and then replace the local bone matrix (12–14). During this process, osteocytes acidify the local microenvironment and secrete factors such as matrix metalloproteases (MMPs) and cathepsin K to resorb local bone matrix, which can be visualized by enlargement and hypomineralization of the lacunae (12, 13, 15, 16), especially in response to lactation. Following weaning, the local bone matrix surrounding osteocytes is replenished.

Maintaining PLR homeostasis is critical as deviations compromise bone quality and increase bone fragility. For example, ablation of transforming growth factor, beta receptor II (*Tgfβr2*) in osteocytes impairs LCN integrity due to suppressed PLR-related gene expression (matrix metalloproteinase 13 (*Mmp13* mRNA), cathepsin K (*Ctsk* mRNA), tartrate resistant acid phosphatases (*Acp5* mRNA)) and increases bone fragility (17). Suppression of PLR not only impairs bone quality but also joint homeostasis. We and others reported signs of PLR suppression following glucocorticoid treatment in mice and in femoral heads from patients with glucocorticoid-induced osteonecrosis (6, 18). These signs include degeneration of the osteocyte LCN, down-regulation of PLR enzyme expression, collagen disorganization, and bone matrix hypermineralization (18). Furthermore, osteocyte-intrinsic ablation of the essential PLR enzyme MMP13 (19) or TGFβR2 (20) in mice suppresses PLR and causes subchondral bone sclerosis and canalicular degeneration. These osteocyte-dependent changes in subchondral bone are sufficient to exacerbate arthritic joint degeneration. Because subchondral bone changes due to PLR suppression may precede rather than follow cartilage degradation, osteocytes could be an ideal target to mitigate joint disease in post-traumatic osteoarthritis or osteonecrosis.

To oppose the effects of suppressed PLR in joint disease in osteoarthritis and osteonecrosis, a potential PLR agonist is parathyroid hormone (PTH). PTH-derived agents are used as bone anabolic therapies and importantly, these agents have proven effective in the treatment of glucocorticoid-induced osteoporosis (21, 22). Among the mechanisms by which PTH induces bone formation, PTH can act directly on osteocytes to suppress SOST expression (23). PTH is also a powerful agonist of osteocyte PLR, especially in lactation (12). This raises the question of whether PTH can rescue skeletal defects in glucocorticoid-treated bone by stimulating osteocytic PLR. We hypothesize that the PLR agonist (PTH(1-34)) can mitigate the effects of GC on the progression of bone and joint disease by restoring suppressed PLR to homeostasis.

To test the hypothesis that a PLR agonist, PTH(1-34), can oppose the suppression of PLR by glucocorticoids, we will evaluate *in vivo* PLR outcomes in a mouse model of glucocorticoid excess treated in the presence or absence of exogenous parathyroid hormone 1-34 (PTH(1-34)). Since suppressed PLR in the subchondral bone is associated with joint disease, the subchondral bone will be assessed using established qualitative and quantitative radiographic, histologic, and molecular approaches. This study aims to uncover the effects of GC and PTH(1-34) on the subchondral bone to guide our understanding of the combined effects of these therapies on the joint.

## 2 Materials and methods

### 2.1 Mouse studies

All animal experiments were approved by the Institutional Animal Care and Use Committee (IACUC) at the University of California, San Francisco. To facilitate comparison to prior work on the role of osteocytes in osteoarthritis, outcomes were analyzed in 16 week old mice (19, 20). Thirteen-week-old male and female FVB/NJ mice (The Jackson Laboratory, #001800, IMSR_JAX:001800) were acclimated to the University of California, San Francisco Laboratory Animal Resource Center (LARC) facility with 67°CF-74°CF, 30-70% humidity, a 12-hr light/dark cycle, and free access to water and irradiated standard chow (LabDiet 5058- PicoLab Rodent Diet 20) for a minimum of two weeks prior to experimental studies (19, 20, 24). At thirteen weeks, mice were randomly assigned for subcutaneous implantation with recommended placebo pellets (Innovative Research of America, cat# NG-111) or slow-releasing prednisolone (GC) pellets (2.1 mg/kg/d, 90-day release, cat# NG-151) for 21 days. Beginning the day after GC pellet implantation, mice received subcutaneous injections (5 days/week) of either vehicle (2% heat-inactivated FBS, 1mM HCl, 150mM NaCl) or rat parathyroid hormone 1-34 (PTH (1–34)) (80 µg/kg; Bachem Cat# H-5460), prior to euthanasia using an IACUC-approved standard procedure of carbon dioxide inhalation at 16 weeks of age.

### 2.2 Micro-computed tomography

Right femurs were dissected free of muscle, fixed in 10% neutral buffer formalin (NBF) for 3 days at 4°C, stored in 70% ethanol and scanned using a Scanco µCT50 scanner with x-ray potential of 55 kVP, current 109 μA, and 6W, at a voxel size (resolution) of 10μm, and 500ms integration time, as previously described (17, 25). Bone structural parameters were analyzed by manually contouring 100 slices of the trabecular (Tb) bone compartment (300µm proximal to epiphyseal plate) below the growth plate or cortical (Ct) compartment at mid-diaphysis using a Scanco analytic software. **Table 1** shows standard µCT parameters (26) for male (n=4-6/group) and female (n=6-7/group) mice.

**Table 1:** Skeletal phenotyping of GC and PTH(1-34) treated male and female mouse bones.

Parameters	Male	GC *(n=6)*	PTH(1-34) *(n=8)*	GC+PTH(1-34) *(n=4)*	Female	GC *(n=7)*	PTH(1-34) *(n=6)*	GC+PTH(1-34) *(n=7)*
	Control *(n=6)*				Control *(n=7)*			
Distal Femur								
Tb. BV/TV	0.125 ± 0.030	0.140 ± 0.012	0.146 ± 0.024	0.145 ± 0.007	0.216 ± 0.037	0.378 ± 0.035**^a,c^**	0.351 ± 0.065**^a,c^**	0.452 ± 0.066**^a^**
Tb. N (1/mm)	4.747 ± 0.352	5.300 ± 0.435	4.806 ± 0.363	5.091 ± 0.359	5.770 ± 1.435	9.125 ± 0.964**^a^**	9.108 ± 1.256**^a^**	10.299 ± 1.002**^a^**
Tb. Th (mm)	0.042 ± 0.006	0.041 ± 0.003	0.047 ± 0.003	0.041 ± 0.003	0.056 ± 0.004	0.062 ± 0.007	0.066 ± 0.006**^a^**	0.068 ± 0.005**^a^**
Tb. Sp (mm)	0.213 ± 0.017	0.189 ± 0.014	0.210 ± 0.015	0.197 ± 0.017	0.185 ± 0.034	0.111 ± 0.012**^a^**	0.114 ± 0.017**^a^**	0.098 ± 0.011**^a^**
Tb. BMD (mg HA/cm^3^)	197.150 ± 34.306	215.651 ±27.243	231.556 ± 36.914	206.775 ± 16.560	281.878 ± 25.99	315.579 ± 20.731**^c^**	309.501 ± 45.272**^c^**	369.269 ± 42.032**^a^**
Tb. TMD (mg HA/cm^3^)	1097.840 ± 45.332	1109.404 ± 13.689	1111.047 ± 18.513	1091.108 ± 28.531	994.096 ± 64.466	835.282 ± 36.099**^a^**	849.375 ± 20.688**^a^**	827.451 ±37.303**^a^**
Midshaft Femur								
Ct. TA (mm^2^)	1.876 ± 0.157	1.917 ± 0.111	1.929 ± 0.127	1.822 ± 0.085	1.754 ± 0.102	1.802 ± 0.128	1.837 ± 0.067	1.854 ± 0.104
Ct. BA (mm^2^)	0.859 ± 0.055	0.840 ± 0.060	0.882 ± 0.117	0.779 ± 0.030	0.934 ± 0.061	0.893 ± 0.097	1.000 ± 0.063**^b^**	0.932 ± 0.020
Ct. BV/TV	0.458 ± 0.015	0.438 ± 0.018	0.456 ± 0.040	0.428 ± 0.014	0.532 ± 0.013	0.495 ± 0.020**^a^**	0.544 ± 0.021**^b,c^**	0.504 ± 0.025**^a^**
Ct. Th (mm)	0.193 ± 0.006	0.185 ± 0.010	0.183 ± 0.040	0.177 ± 0.005	0.219 ± 0.004	0.194 ± 0.016**^a^**	0.216 ± 0.011**^b,c^**	0.189 ± 0.013**^a^**
Ct. BMD (mg HA/cm^3^)	698.210 ± 20.397	672.129 ± 45.756	704.795 ± 73.954	643.283 ± 19.773	722.743 ± 16.272	654.762 ± 30.918**^a^**	734.263 ± 25.539**^b,c^**	663.662 ± 37.091**^a^**
Ct. TMD (mg HA/cm^3^)	1455.383 ± 30.407	1462.964 ± 37.229	1473.442 ± 28.144	1443.766 ± 28.680	1355.346 ± 14.940	1339.512 ± 10.523	1357.629 ± 6.483**^c^**	1326.397 ± 17.495**^a^**

Bone parameters on 16 week old male and female right femurs that were measured by µCT include trabecular (Tb.) and cortical (Ct.) parameters on the distal femoral and mid-shaft femur regions, respectively. Trabecular parameters were reported as: Trabecular bone volume fraction (Tb. BV/TV), Trabecular number (Tb. N), Trabecular thickness (Tb. Th), Trabecular separation (Tb. Sp), Trabecular bone mineral density (Tb. BMD), Trabecular tissue mineral density (Tb. TMD). Cortical parameters were reported as: Cortical total area (Ct. TA), Cortical bone area (Ct. BA), Cortical bone volume fraction (Ct. BV/TV), Cortical thickness (Ct. Th), Cortical bone mineral density (Ct. BMD), Cortical tissue mineral density (Ct. TMD). Data are presented as mean ± SD with **^a^** p ≤ 0.05 statistically different from Female Control group, **^b^** p ≤ 0.05 statistically different from Female GC group, **^c^** p ≤ 0.05 statistically different from Female GC+PTH(1-34) group. Statistical differences were determined with two-way ANOVA with post-hoc Holm Sidak.

### 2.3 Flexural strength tests/three-point bending test

Unfixed left femurs (n=4-8/group) were subjected to three-point bending at mid-shaft to assess mechanical properties using a Bose Electroforce 3200 (RRID: SCR_019752) test frame (27). Briefly, bones were hydrated in 1X phosphate-buffered saline (PBS) at room temperature and placed on 2 lower supporting jigs (8mm apart) with the anterior side facing down. The test probe was placed at the mid-point between the 2 supporting jigs to create bending with a displacement rate of 10 µm/s. Mechanical properties of stiffness, yield force, and ultimate force were calculated from load-displacement curves using a custom MATLAB (RRID: SCR_001622) script as previously described (27, 28). Material properties of elastic modulus, yield stress, ultimate stress was calculated from µCT measurements of left femurs from 16-week-old male (n=2-7/group) and female (n=5-6/group) mice using the femur cross-section diameter and moment of inertial (Imin/Cmin and Imin) and equations from Turner et al. and Jepsen et al. (28, 29).

### 2.4 Nanostring nCounter analysis

RNA was extracted from female humeri (n=4 mice/group) after removal of epiphysis and bone marrow to assess transcriptomic profiles of osteocyte-enriched cortical bone. Briefly, the dissected bones were flash frozen in liquid nitrogen and homogenized in QIAzol Lysis Reagent (Qiagen cat #79306), and total RNA was extracted using the RNeasy mini kit (Qiagen cat#74106) according to the manufacturer’s instructions. Direct mRNA counts were determined using an automated Nanostring nCounter Mx system (RRID: SCR_021712) (30, 31) with a custom probe set for 94 mouse skeletal genes in the UCSF CCMBM Skeletal Biology and Biomechanics Core. Analysis of expression profiles was performed using the nSolver Analysis Software (RRID: SCR_003420) and nCounter Advanced Analysis Software and normalized with seven housekeeping genes (*Gapdh, Rpl19, Gilz* (*Tsc22d3*), bone sialoprotein (*Ibsp*), beta-2 microglobulin (*B2m*), beta actin (*Actb*), *Serpine2*). Highly significant gene expression fold changes were determined by unpaired t-tests between experimental groups.

### 2.5 Cell culture

Osteocyte-like MLO-Y4 cells (provided by L. Bonewald, RRID: CVCL_M098) were maintained in alpha-MEM supplemented with 2.5% fetal bovine serum, 2.5% bovine calf serum, and 1% penicillin-streptomycin and grown on rat tail collagen type 1 (0.16 mg/ml) coated plates. MLO-Y4 cells were treated with 0.1µM or 1µM dexamethasone with or without 50 nM rat parathyroid hormone 1-34 [PTH(1-34)] for 24 hours (n=3 biological replicates/group and 2 independent experiments). RNA was extracted for real-time quantitative PCR (qPCR), using iQ SYBR Green Supermix (BioRad) on a Biorad CFX96 Touch Real-Time PCR Detection System (RRID: SCR_018064). Gene expression levels were normalized to the housekeeping gene *Gapdh*. Additional details for primers are provided in the **Supplementary Table 1**. Fold change was determined using the delta-delta CT method (32). A one-way ANOVA was used for statistical analysis.

### 2.6 Histology

Female right femur/tibia joints were dissected free of muscle, fixed in 10% neutral buffered formalin (NBF), decalcified in 10% EDTA, dehydrated, and embedded with knee joints positioned at a 45 angle in paraffin as previously described (19, 20). Coronal sections (7μm) of the knee joints were obtained using a microtome (Leica Microsystems, Buffalo Grove, IL), followed by standard dewaxing and hydration protocols (19, 20) before various histological staining described below. All brightfield images were obtained on a Nikon Eclipse E800 microscope (RRID: SCR_020326).

### 2.7 Safranin O/fast green and OA scoring

Knee joints sections were stained with the Safranin O/Fast Green using the protocol adapted from University of Rochester (33) with the following modifications: Weigert’s Iron Hematoxylin incubation for 3 mins, brief water rinse and differentiation in 1% acid-alcohol for 15 secs, stain with 0.02% Fast Green for 5 mins, differentiation with 1% acetic acid for 30 secs, rinse with water and incubation in 1% Safranin-O for 10 mins, prior to mounting with mounting media.

Osteoarthritis scoring of Safranin O/Fast Green-stained coronal sections (n=4/group) was performed by three blinded graders using the OARSI (34) and modified Mankin (35) scoring system. To maintain a consistent region of interest of the knee, sections with visible anterior cruciate ligament (ACL) and posterior cruciate ligament (PCL) were used for grading. Quantification of the whole knee joint was obtained using 10X and stitched 20X images to assess each quadrant of the knee joint (femur, tibia, lateral, medial). Mean scores across all blinded graders were obtained and the mean scores were averaged within each experimental group.

### 2.8 Tartrate-resistant acid phosphatase stain

Bone resorption activity in the knee joint was observed using the tartrate-resistant acid phosphatase (TRAP) Leukocyte Acid Phosphatase staining kit (Sigma cat# 387) following the manufacturer’s instructions with slight modifications. Briefly, sections were post-fixed for 30 secs in Fixative Solution, rinsed in water, and incubated with a mixture of Fast Red Violet (Sigma cat#F3381) and Fast Garnet GBC Base Solution for 1 hour at 37°C in the dark. Slides were then rinsed in water and counterstained with 0.02% Fast Green (Sigma cat# F3381) and mounted. For quantification of bone resorption parameters, one image (20X) of the subchondral bone per quadrant of the knee joint (femur, tibia, medial, lateral) was evaluated. A total of 4 images per animal (n=4-5 mice/group) were analyzed by a blinded grader using the open source image analysis software TrapHisto (36) to measure the Osteoclast Surface per Bone Surface (Oc.S/BS %) and the Number of Osteoclasts per Tissue Volume (N.Oc/TV mm^-2^). The mean of these parameters was averaged per quadrant of the knee for each animal and averaged within each experimental group to acquire mean total, medial and lateral joint values.

### 2.9 Ploton silver nitrate stain

The lacunocanalicular network of the subchondral bone in the knee was visualized by Ploton silver nitrate stain as previously described (17, 19, 20, 37). Briefly, right knee joint sections were stained in a fresh mixture of 50% silver nitrate and 1% formic acid in 2% gelatin with a 2:1 ratio for 55 mins in the dark and then counterstained with Cresyl Violet. For consistency, sections with visible ACL and PCL were chosen for staining. Four high-resolution images (100X) per knee joint subchondral bone quadrant (femur, tibia, medial, lateral) were used for quantitative analysis. ImageJ (RRID: SCR_003070) was used by a blinded grader to quantify lacunar number and lacunae size for a total of sixteen images per animal (n=4 mice group) by converting to a binary image, manually contouring each lacunae, and measuring with the Analyze Particles feature. Mean values were obtained per quadrant of the knee per animal and were then averaged within each experimental group.

### 2.10 Statistical analysis

All data are represented as mean ± standard deviation (SD) or standard error mean (SEM) as appropriate for each assay, as stated in the figure legends. For *in vivo* data, the number of samples per group is denoted as “n”, while *in vitro* data, n indicates the number of independent experiments/biological replicates. GraphPad Prism (GraphPad Software version 10) was used for all statistical analysis and statistical significance required a p-value ¾ 0.05.

## 3 Results

### 3.1 Dimorphic effects of GC and PTH (1–34) on bone structure and mechanics

**Figure 1:**
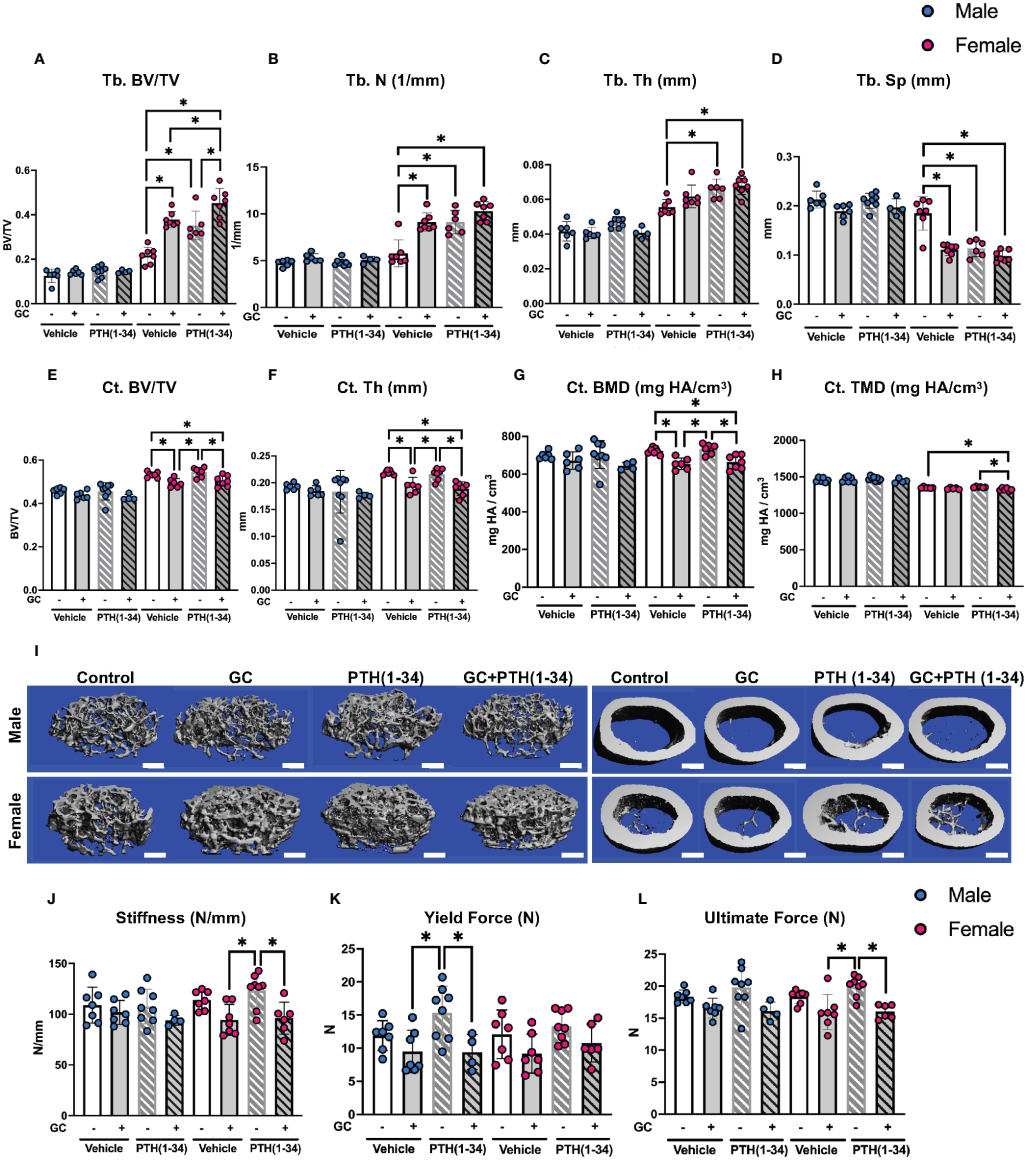
GC and PTH(1-34) effects on bone quantity and quality are sexually dimorphic. Femora of 16-week-old control and GC and/or PTH(1-34) treated male (n=4-8/group) and female (n=6-7/group) mice were analyzed using µCT for trabecular (Tb.) **(A–D)** and cortical (Ct.) parameters **(E–H)** on distal femur and mid-femur respectively. Results reveal trabecular bone/volume fraction (Tb. BV/TV, **A**), trabecular number (Tb. N, **B**), trabecular thickness (Tb. Th, **C**), trabecular separation (Tb. Sp, **D**), cortical bone volume fraction (Ct. BV/TV, **E**), cortical thickness (Ct. Th, **F**), cortical bone mineral density (Ct. BMD, **G**), and cortical tissue mineral density (Ct. TMD, **H**). Representative µCT reconstructions display sexual dimorphism (scale bar = 500µm) **(I)**. Three-point bending on male (n=4-8/group) and female (n=6-8/group) left femora show outcomes of flexural strength **(J–L)**. In each graph, male data is displayed as blue dots, with female data displayed as red dots. Data are presented as mean ± SD. Statistically significant differences (*p≤0.05) were determined by two-way ANOVA with *post-hoc* Holm Sidak within sex.

Micro-computed tomography (µCT) identified sex-dependent differences in the effect of GC, PTH(1-34), and combined GC+PTH(1-34) treatments on bone phenotypes (**Figure 1**; **Table 1**). At 16 weeks of age, male mice, regardless of treatment type, showed no significant changes in either trabecular (Tb) (**Figures 1A–D**) or cortical (Ct) (**Figures 1E–H**) bone parameters by the drug treatments versus vehicle controls, as visualized in the 3D-reconstructed images (**Figure 1I**) and their quantifications (**Figures 1A–H**). In contrast, female mice treated for 21 days with GC showed significant increases in Tb fraction (Tb.BV/TV) (**Figure 1A**) and number (Tb.N, **Figure 1B**), with a complementary decrease in spacing (Tb.Sp, **Figure 1D**). GC treatment caused loss of Ct bone in female mice (**Figures 1E–H**), similar to what we and others previously reported (6, 18, 38, 39), revealing the trabecular versus cortical region-specific effects of GC. In 16 week old female mice, intermittent PTH (1–34) treatment caused the anticipated anabolic response with significantly elevated Tb.BV/TV (**Figure 1A**), Tb.N (**Figure 1B**), and Tb.Th (**Figure 1C**), and reduced Tb.Sp (**Figure 1D**). Combined GC and PTH(1-34) treatment significantly increased Tb bone parameters relative to female controls (**Figures 1A–D**), with even greater increases in Tb.BV/TV than each treatment alone (**Figure 1A**). However, combined GC and PTH(1-34) did not mitigate GC-induced Ct bone loss (**Figures 1E–H**).

Mechanical testing by three-point bending showed that male femurs treated with PTH(1-34), relative to those treated with GC, have significantly increased yield force, but this effect is absent when GC and PTH(1-34) are combined (**Figures 1J–L**). Similar trends are present in females, with PTH-dependent increases in stiffness and ultimate force relative to bone from GC-treated mice (**Figures 1J–L**). As in males, PTH(1-34) does not overcome the effect of GC on mechanical properties in female bone (**Table 2**). Material properties of male or female bones were unaffected by GC or PTH(1-34) (**Figure 1**).

**Table 2:** Mechanical and Material properties of GC and PTH(1-34) treated male and female mice.

Flexural Strength Parameters	Male	GC *(n=7)*	PTH(1-34) *(n=8)*	GC+PTH(1-34) *(n=4)*	Female	GC *(n=7)*	PTH(1-34) *(n=8)*	GC+PTH(1-34) *(n=6)*
	Control *(n=7)*				Control *(n=7)*			
Stiffness (N/mm)	108.927 ± 17.683	101.823 ± 11.896	107.614 ± 17.106	94.115 ± 4.989	113.855 ± 9.294	94.414 ± 15.243	123.528 ± 16.690**^b,c^**	96.115 ± 15.722
Yield Force (N)	11.887 ± 2.254	9.490 ± 3.205	15.358 ± 3.992**^#,$^**	9.405 ± 2.637	12.089 ± 3.675	9.180 ± 2.943	13.311 ± 2.374	10.788 ± 2.860
Ultimate Force (N)	18.383 ± 0.996	16.581 ± 1.545	19.771 ± 3.214	16.075 ± 1.431	18.336 ± 1.040	15.960 ± 2.736	19.969 ± 1.599**^b,c^**	16.038 ± 1.106

Material Property Parameters	Male	GC *(n=5)*	PTH(1-34) *(n=7)*	GC+PTH(1-34) *(n=2)*	Female	GC *(n=5)*	PTH(1-34) *(n=6)*	GC+PTH(1-34) *(n=5)*
	Control *(n=6)*				Control *(n=6)*			
Elastic Modulus (MPa)	117339.527 ± 87134.996	104138.674 ± 100265.754	122768.693 ± 93711.133	69529.000 ± 65118.411	111678.620 ± 81807.547	142092.636 ± 112922.939	945682.682 ± 67164.682	104878.108 ± 111369.976
Yield Stress (MPa)	590.547 ± 336.268	439.776 ± 501.112	717.127 ± 457.459	430.620 ± 414.944	608.432 ± 432.996	628.032 ± 537.062	551.870 ± 396.676	553.454 ± 666.289
Ultimate Stress (MPa)	925.777 ± 551.046	635.606 ± 517.754	974.986 ± 670.455	642.100 ± 466.959	896.548 ± 561.744	1129.804 ± 801.744	782.733 ± 466.431	728.002 ± 658.929

Flexural strength test of right femurs of 16 week old male and female mice were performed by three-point bending. Outcomes on femurs are reported as Stiffness (N/mm), Yield Force (N), and Ultimate Force (N). Material Properties are reported as Elastic Modulus (MPa), Yield Stress (MPa), and Ultimate Stress (MPa). Data are presented as mean ± SD with **^#^** p ≤ 0.05 statistically different from Male GC group, **^$^** p ≤ 0.05 statistically different from Male GC+PTH(1-34) group. **^b^** p ≤ 0.05 statistically different from Female GC group, **^c^** p ≤ 0.05 statistically different from Female GC+PTH(1-34) group. Statistical differences were determined with two-way ANOVA with post-hoc Holm Sidak.

### 3.2 GC and PTH(1-34) regulation of genes implicated in bone resorption

**Figure 2:**
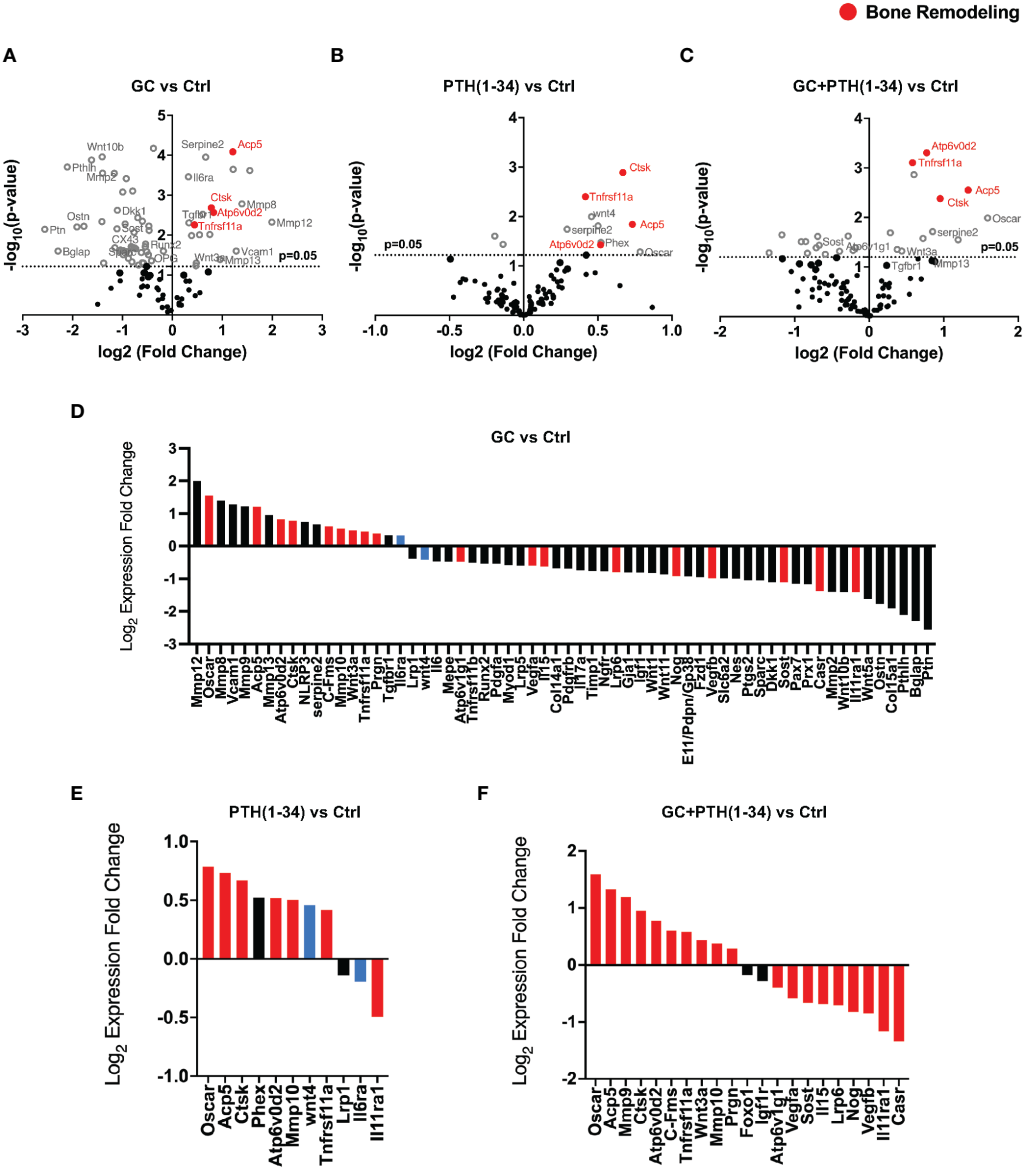
GC and PTH(1-34) effects on skeletal gene expression in female cortical bone. Volcano plots of 96 skeletal-associated mouse genes from Nanostring analysis shows significantly up- and down-regulated genes associated with bone remodeling (red dots) in treated (GC, PTH(1-34), and GC+PTH(1-34)) female mice (n=4) compared to controls **(A–C)**. Statistically expressed genes (gray dots) are above the horizontal p-value threshold (dotted gray line) and up-regulated or down-regulated genes fall to either to the right or left sides, respectively. Highly significantly gene expression fold changes was determined by unpaired t-test between experimental groups, normalized to 7 housekeeping genes (*Gapdh, Rpl19, Gilz* (*Tsc22d3*), bone sialoprotein (*Ibsp*), beta-2 microglobulin (*B2m*), beta actin (*Actb*), *Serpine2*). **(D–F)** show statistically up- or down-regulated genes in each condition, with red bars indicating genes that are regulated in the same manner as combined GC+PTH(1-34) treatment, and blue bars indicating genes that are opposingly regulated between GC and PTH(1-34) treatment.

We evaluated the effect of GC, PTH(1-34), and GC+PTH(1-34) treatment on gene expression from osteocyte-enriched humeri using Nanostring nCounter assay and a custom probe set of 96 mouse genes important in skeletal biology, including bone, cartilage, tendon, and muscle. By directly measuring mRNA, this assay provides increased sensitivity across a range of conditions (**Supplementary Figures 2A–H**). Volcano plots show regulation of several genes associated with bone remodeling in osteocyte-enriched bones across all treatment groups from female (**Figures 2A–C**) and, to a lesser extent, from male mice (**Supplementary Figure 1**). We previously reported that a 7-day GC treatment downregulates *Mmp2* (18), which is recapitulated with 21-day treatment of GC (**Figure 2D**). In addition, as anticipated based on prior reports (38, 40), GC reduced mRNA levels of osteocrin (*Ostn*), osteoprotegerin (*Tnfrsf11b*), gap junction alpha 1 protein (*Cx43*) (*Gja1*), while increasing mRNA levels for tartrate resistant acid phosphatase (*Acp5*) and cathepsin K (*Ctsk*) (**Figure 2D**), confirming the efficacy of GC in these conditions. We previously reported that a 7-day GC treatment suppressed bone remodeling genes implicated in PLR (18), however here we observe that a longer 21-day GC treatment significantly upregulates several PLR-related genes including *Acp5, Mmp13, Atp6v0d2, Ctsk* (**Figure 2D**).

As expected based on prior reports of PTH(1-34) induction of *Phex* (41, 42) and *Wnt4* (43), both genes are enriched in bone from the PTH (1–34) treated group (**Figure 2E**). Other PTH(1-34)-suppressed genes (*Sost*, *Dmp1, Osteocalcin*) (44–46) and PTH(1-34)-induced genes *(Tnfrsf11a* (*Rank), Tnfrsf11b (Opg*)) (43, 47) were not differentially expressed in these conditions. As we had hypothesized, PTH(1-34) also increased mRNA levels for several PLR-related genes (*Acp5, Ctsk, Atp6v0d2*), as well as *Tnfrsf11a (Rank)* (**Figure 2E**). The combined GC + PTH(1-34) treatment led to upregulation of *Tnfrsf11a* and the same PLR-related genes (*Acp5, Ctsk, Atp6v0d2*) as individual treatments (**Figure 2F**). Indeed, of the 21 genes in this panel that are significantly regulated by GC+PTH(1-34), relative to vehicle treated cells, all but 2 (*Foxo1* and *Igf1r*) are regulated in the same manner by GC or PTH(1-34) alone, with 7 regulated by both stimuli (**Figures 2D–F**, red bars). Overall, analysis of gene expression in these conditions suggests that GC and PTH(1-34), alone or combined, shift bone toward a more catabolic state.

### 3.3 Osteocyte-intrinsic suppression of MMP13 by GC is not rescued by PTH(1-34)

**Figure 3:**
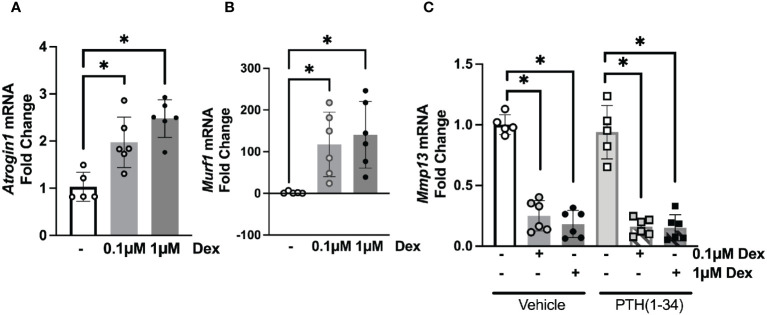
Osteocyte-intrinsic suppression of *Mmp13* by GC is not rescued by PTH(1-34). Real-time qPCR analysis on MLO-Y4 cells treated with low (0.1µM) or high (1µM) dose of Dexamethasone (DEX) causes induction of *Atrogin1* **(A)**, *Murf1* **(B)** and dose-dependent down-regulation of *Mmp13* **(C)** mRNA (n=3 replicates/group and 2 independent experiments) normalized to GAPDH. PTH(1-34) did not mitigate effects of GC treatment on *Mmp13* **(C)**. Data is displayed as mean ± SD and statistically significant differences (*p≤0.05) were determined using one-way ANOVA.

To determine the direct actions of GC and PTH(1-34) on osteocytic activities, we cultured osteocyte-like MLO-Y4 cells with dexamethasone (DEX) with or without PTH(1-34) for 24 hours prior to RNA isolation. Real-time qPCR analysis confirmed the dose-dependent (0.1µM and 1µM) effects of DEX on glucocorticoid-inducible *Atrogin1* and *Murf1* gene expression (**Figures 3A, B**). Consistent with the previously reported DEX-dependent decrease in *Mmp13* mRNA levels in cultured osteocytes (18), DEX suppresses *Mmp13* expression in an osteocyte-intrinsic manner (**Figure 3C**). This result suggests that other osteocyte-independent factors may counteract the direct actions of GC on osteocytes to increase *Mmp13* expression in osteocyte-enriched cortical bone *in vivo* (**Figure 2**). PTH(1-34) did not mitigate suppression of *Mmp13* expression by DEX (**Figure 3C**). These *in vitro* experiments along with the above *in vivo* studies highlight both cell-intrinsic and non-autonomous actions of GC and PTH(1-34) on osteocytes, and the inability of PTH(1-34) to rescue downregulated *Mmp13* expression of GC on osteocytes.

### 3.4 GC and PTH(1-34) regulation of articular cartilage and subchondral bone homeostasis

**Figure 4:**
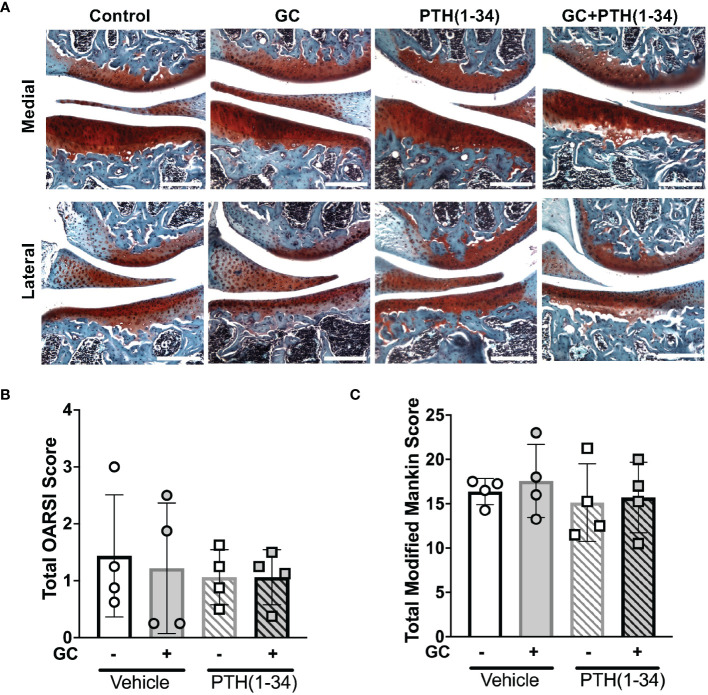
Joint and osteoarthritis assessment of GC and PTH(1-34) treated females. Safranin O/Fast Green stain of right knee joints from 16-week-old control and GC and/or PTH(1-34) treated females (n=4/group) show no changes in cartilage (red) and subchondral bone (counterstain blue/green) knee joint phenotypes in representative images (20X, scale bar = 200µm) **(A)**, supported by quantified total OARSI **(B)** and total Modified Mankin Score **(C)**. Data are presented as mean ± SEM and statistically significant differences were determined by two-way ANOVA with *post-hoc* Holm Sidak between experimental groups.

Given that several of the GC and PTH(1-34) regulated genes can participate in bone resorption executed by either osteoclasts or osteocytes (12, 13, 48, 49), both of which can impact joint homeostasis (19, 50, 51), we sought to determine the effect of these treatments on articular cartilage and subchondral bone. Since microCT (µCT), mechanical testing, and gene expression analysis show greater sensitivity to GC and PTH(1-34) in females in these conditions, the remainder of this study focuses on female mice. The effect of GC and PTH(1-34) on the joint was evaluated in Safranin O/Fast green stained knee joint sections (**Figure 4A**) using standard OARSI (**Figure 4B**) (34) and modified Mankin Score (**Figure 4C**) grading systems (35). Across treatments, no signs of cartilage damage or early onset osteoarthritis were observed in 16-week-old female mice.

**Figure 5:**
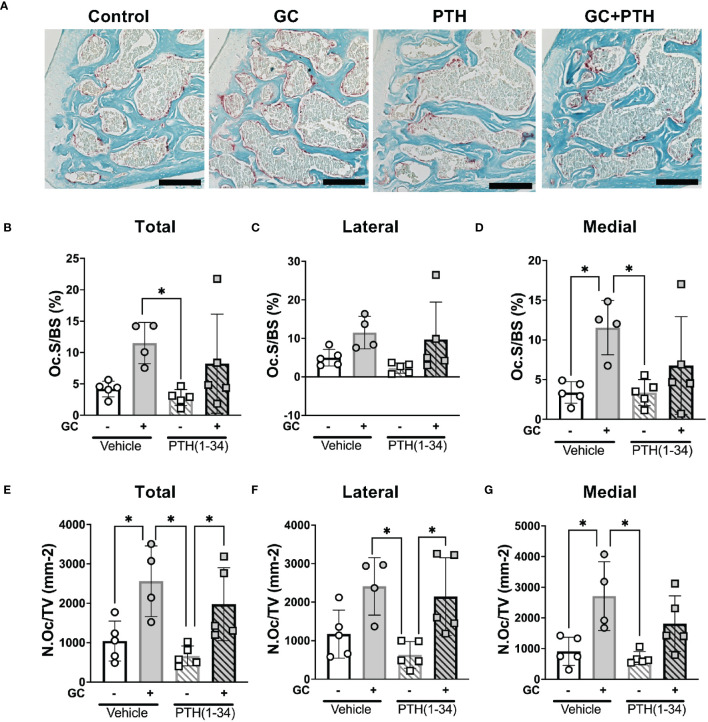
Effects of GC and PTH(1-34) on TRAP activity. TRAP staining on subchondral knee sections of control and treated (GC, PTH(1-34), or GC+PTH(1-34)) 16-week-old female mice (n=4-5/group). Representative images from each condition (**A** 20X, scalebar = 200 µm) provide visualization of TRAP+ stained cells (red), counterstained in methyl green. Quantification of Osteoclast Surface per Bone Surface (Oc.S/BS %) and Number of Osteoclasts per Tissue Volume (N.Oc/TV mm^-2^) were analyzed in each joint compartment (femur, tibia, medial, lateral) and displayed as total **(B, E)**, lateral **(C, F)**, and medial **(D, G)**. Data are presented as mean ± SD and statistically significant differences (*p≤0.05) were determined by two-way ANOVA with *post-hoc* Tukey was performed between experimental groups.

Among the catabolic genes induced by GC, PTH(1-34), and GC+PTH(1-34) is *Acp5 (Trap)*, which can be expressed by osteoclasts or by osteocytes engaged in PLR (12, 52). TRAP staining was used to distinguish the cell populations associated with differential *Acp5/Trap* expression in subchondral bone of the female mouse knee (**Figure 5**; **Table 3**). While abundant TRAP staining was detected on the surfaces of bony trabeculae, corresponding to osteoclasts (**Figures 5B–D**), relatively few TRAP-positive osteocytes were detected in any condition (**Figure 5A**). Quantitative analysis of the % osteoclast surface per bone surface (Oc.S/BS %) (**Figure 5D**) and number of osteoclast per tissue volume (N.Oc/TV mm^-2^) (**Figure 5G**) revealed that GC significantly elevated TRAP activity in the medial subchondral bone, which contributed to the increase in total subchondral bone TRAP activity (**Figures 5B, E**). TRAP activity was unaltered by PTH(1-34) alone or in combination with GC (**Figures B–G**). The inability of PTH(1-34) to oppose GC-induced TRAP activity is consistent with their shared trabecular bone phenotype and *Acp5* expression profile.

**Table 3:** Bone resorption parameters of GC and PTH(1-34) treated female mice.

Bone Resorption Parameters	Female	GC *(n=4)*	PTH(1-34) *(n=5)*	GC+PTH(1-34) *(n=5)*
	Control *(n=5)*			
Total				
Oc.S/BS (%)	4.180 ± 2.405	11.517 ± 4.091	2.827 ± 1.714**^b^**	8.235 ± 7.717
N.Oc/TV (mm^-2^)	1041.311 ± 617.545	2561.002 ± 1157.082**^a^**	661.324 ± 357.464**^b,c^**	1976.699 ± 927.426
Lateral				
Oc.S/BS (%)	5.068 ± 2.471	11.488 ± 4.577	2.013 ± 1.481	8.429 ± 9.293
N.Oc/TV (mm^-2^)	1120.549 ± 662.070	2409.868 ± 987.548	556.028 ± 379.503**^b,c^**	1900.331 ± 975.607
Medial				
Oc.S/BS (%)	3.382 ± 2.167	11.546 ± 3.862**^a^**	3.363 ± 1.835 **^b^**	6.769 ± 5.882
N.Oc/TV (mm^-2^)	911.490 ± 573.854	2712.136 ± 1357.012**^a^**	690.619 ± 351.894**^b^**	1810.291 ± 895.802

Tartrate-resistant acid phosphatase (TRAP) activity of the right knee subchondral bone regions of 16 week old female mice was detected by TRAP staining. Quantification on TRAP stains are reported as: Osteoclast Surface (Oc.S), Bone Surface (BS), Number of Osteoclasts (N.Oc) and Tissue Volume (TV). Data are presented as mean ± SD with **^a^** p ≤ 0.05 statistically different from Control group, **^b^** p ≤ 0.05 statistically different from GC group, **^c^** p ≤ 0.05 statistically different from GC+PTH(1-34) group. Statistical differences were determined with two-way ANOVA with post-hoc Tukey.

Both GC and PTH(1-34) regulate osteocytic PLR (12, 18) and the expression of genes implicated in this process, including *Mmp13, Atp6v0d2*, and *Ctsk*, as shown previously (13, 18, 53) and in **Figure 2**. Disruption of the osteocyte lacunocanalicular network (LCN) is a hallmark of PLR suppression that results from GC treatment (18) or from osteocytic ablation of *Mmp13* or *Ctsk* (13, 19, 49). In addition, long-term GC exposure induces osteocyte apoptosis (54, 55). Therefore, to examine the effect of GC and PTH(1-34), alone or in combination, on subchondral bone, osteocyte apoptosis and the LCN were examined histologically using terminal deoxynucleotidyl transferase dUTP nick end labeling (TUNEL) and Ploton silver nitrate stain, respectively. Though some apoptotic marrow cells, osteoclasts, and osteocytes were detected in each condition, the number of TUNEL-positive osteocytes was low and unchanged by GC or PTH(1-34), alone or in combination (**Supplementary Figure 3**).

**Figure 6:**
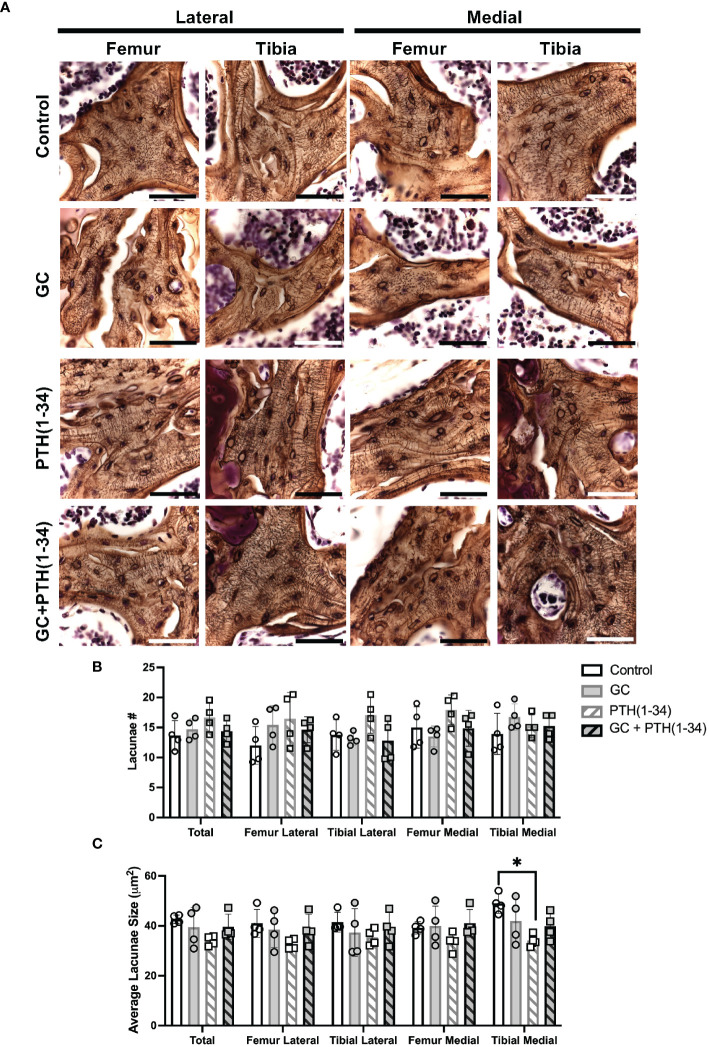
Subchondral bone assessment of GC and/or PTH(1-34) treated female mouse knees. Representative high-resolution images (100X, scale bar = 50 µm) of the right knee joints of control and treated (GC, PTH(1-34), or GC+PTH(1-34)) females at 16-week-old (n=4/group) stained with Ploton silver nitrate stain and counterstained with Cresyl Violet show the subchondral bone lacunocanalicular network (LCN) **(A)**. Quantitative analysis of the number (#) of lacunae **(B)** and average lacunae size **(C)** shows treatment effects on the LCN in each joint compartments (femur, tibia, medial, lateral). Data are presented as mean ± SD, and statistically significant differences (*p≤0.05) were determined by unpaired t-test between experimental groups.

Silver staining permits qualitative analysis of canalicular organization (**Figure 6A**) and quantification of lacunar number (**Figure 6B**) and lacunae size (**Figure 6C**) were quantified in each subchondral bone quadrant of the knee. Unlike cortical bone, canalicular organization in trabecular bone is more variable, such that treatment-specific differences in canalicular integrity were not apparent. While GC-dependent differences in lacunar number or size were not observed, PTH(1-34) treatment showed the greatest effect on increased lacunar number in the femur medial compartment (**Figure 6B**) and decreased lacunar size in the tibia medial compartment (**Figure 6C**). The elevated number of lacunae and reduced average lacunar size observed with PTH(1-34) treatment is mitigated when combined with GC. This demonstrates that GC and PTH(1-34) effects on the osteocyte LCN in these conditions are mild, and that the modest effect of PTH(1-34) on lacunar size is blocked by exogenous GC.

## 4 Discussion

Given the central role of GC and PTH as powerful endocrine regulators, as well as their widespread therapeutic use, this study advances the field by examining their combined effects on clinically relevant markers of osteocyte function in the context of bone and joint health. GC is a well-established risk factor for osteonecrosis (56) that affects multiple cell types, including osteoblasts, osteoclasts, and osteocytes (6, 55, 57–62). We previously showed evidence of osteocyte PLR suppression in subchondral bone of humans and mice following glucocorticoid treatment (18). Since PTH can stimulate PLR (12, 23, 63), we sought to determine whether PTH(1-34) can oppose the suppression of osteocytic PLR by glucocorticoids in subchondral bone. We examined tissue, cellular, and molecular outcomes in bone from mice treated with or without glucocorticoid, in the presence or absence of parathyroid hormone 1-34. Although prior studies suggested that PTH might be sufficient to reverse some effects of GC on osteocyte PLR, we find that PTH(1-34) either has no effect or exacerbates the catabolic effects of GC on bone in these conditions.

The effects of PTH(1-34) and GC on the skeletal phenotype are sensitive to the treatment dose and timing, and to mouse age, sex, and strain. Here, PTH(1-34) was administered a day after GC pellet implantation, when these two treatments may be antagonizing each other. Others have also observed attenuated anabolic effects of PTH(1-34) or abaloparatide, a parathyroid hormone-related peptide analog, in the presence of GC (24, 64, 65). PTH(1-34) may have shown a more robust effect if its administration after GC pellet implantation was delayed. For example, the loss of trabecular bone and decreased bone quality in GC-treated Swiss-Webster mice was restored by PTH(1-34) that was administered 28 days post-GC treatment (4). Optimal anabolic effects were reported in male mice treated with PTH of 30-60 µg/kd/day for 5-6 weeks beginning after 12 weeks of age (66). Treatments in this study commenced at 13 weeks of age and continued for 3 weeks with a higher dose of PTH(1-34) of 80 µg/kd/day. Greater anabolic effects of PTH(1-34) treatment may have been observed if treatment length was extended beyond 21 days and if PTH(1-34) treatment was delayed post-GC pellet implant.

Here we observe sexual dimorphism in the skeletal response to GC and PTH(1-34) treatment, where female mice are more sensitive to GC and PTH(1-34) compared to males. GC is known to have dimorphic effects, such that female mice are more sensitive to glucocorticoid-induced muscle atrophy (67), possibly due to differences in how GC is metabolized (68). In our study, GC induces more trabecular bone formation and cortical bone loss in female mice, highlighting GC’s region-dependent effects on the bone phenotype. Similar sex-specific differences were previously reported in C57BL/6 mice treated with prednisolone, with females more sensitive to glucocorticoid induced cortical bone loss and fragility than males (69). Although the increased trabecular bone may seem contrary to the well-defined GC-induced bone loss (70), the effects of GC on bone are sensitive to many factors, including the background strain of the mice (70–72), age, and dosing regimen. Other studies report elevated trabecular bone in female mice (73) and unaltered trabecular bone in the lumbar vertebrae of male rats (74). This study used FVB mice, which are the most susceptible strain to study GC-induced osteonecrosis, but at 13-weeks of age, they may be less sensitive to the catabolic action of GC on trabecular bone. Indeed, the effects of GC are age-dependent, such that others have shown that GC’s effect on trabecular bone is unchanged (70, 72, 75, 76) or elevated (75) in younger mice. Another variable to consider is GC dosing effects, as shorter exposure to higher dose GC (77) or prolonged lower dose GC (40) treatment in younger mice can cause bone loss. As expected, PTH(1-34) effects on the skeletal phenotype also show sexual dimorphism (78, 79), where females are more sensitive to PTH(1-34) than males. The anabolic effects of PTH(1-34) on trabecular and cortical bone in females are blocked in the presence of GC. An increase in cortical porosity may contribute to the effect of PTH(1-34) on microCT (µCT) and mechanical outcomes observed here (80). Collectively, these studies highlight the critical role of biological variables in determining the effects of GC and PTH(1-34) on the skeleton, including age, sex, dose, and duration of the treatments.

Sexual dimorphic effects of glucocorticoid excess have also been observed in humans. For example, males with Cushing’s syndrome, a condition with elevated glucocorticoid exposure, are more susceptible to osteoporosis, while females experience more metabolic symptoms such as hyperglycemia, obesity, and hyperlipidemia (68). On the other hand, female liver transplant patients on chronic glucocorticoid therapy have a higher risk of fracture than males (81). Other rodent studies show sexually dimorphic responses to glucocorticoids in metabolism (68, 82), inflammation (83, 84), skeletal muscle (85), stress responses (86), and liver, heart, and adipose tissues (68), all of which can exert primary or secondary effects on bone. The mechanisms by which glucocorticoids cause sexually dimorphic skeletal responses require further study.

Our prior studies supported the conclusion that GC suppressed PLR through osteocyte-intrinsic suppression of genes required for resorption of the peri-osteocytic bone matrix, such as *Mmp13* (18). Although the current study also shows GC-dependent repression of *Mmp13* mRNA levels in cultured osteocytes, prolonged treatment of GC increases mRNA levels for *Mmp13*. In addition, GC treatment of female mice for 21 days increased levels of many other catabolic genes in cortical bone, including *Ctsk, Acp5, Tnfrsf11a, Atp6v0d2.* Since these genes participate in bone resorption by both osteoclasts and osteocytes, it was unclear which cell type was the target of GC effects on gene expression. We observed significant changes in osteoclast TRAP activity, but the osteocyte-intrinsic effects of GC in this study are insufficient to explain the effect of GC on cortical bone gene expression, and may relate to acute vs. chronic effects of GC. Importantly, PTH(1-34), alone or in combination with GC, did not mitigate the induction of catabolic genes. Similar results were observed when GC blunted effects of the PTHrP analog, abaloparatide, on femoral bone mass and strength (24). These molecular findings support the tissue-level conclusions that PTH(1-34) does not oppose the effects of GC in osteocytes.

The recovery of bone following elevated glucocorticoid exposure has been examined in many clinical and preclinical studies. Following discontinuation of glucocorticoid use, patients have shown full (87) or partial recovery of bone mineral density bone (88) and decreased fracture risk (89, 90). Patients with Cushing’s disease show recovery of bone mineralization after 6 months of disease remission, with fracture risk decreasing to baseline levels in controls after 9-15 months (91). Despite recovery of bone density and fracture resistance, the effects of glucocorticoids on bone material properties remain (91). Supporting the persistent effects of glucocorticoids on bone, within 3 months after glucocorticoid withdrawal, rats showed partial recovery of bone loss but still have impaired bone quality (92). A better understanding of the reversibility of glucocorticoid effects on bone quality is especially relevant for glucocorticoid-induced osteonecrosis (18, 93, 94), and for post-menopausal women with long-term glucocorticoid use, whose risk of vertebral fractures is higher than expected based on their bone mineral density (58, 95).

Pathological changes in subchondral bone structure, mechanics, and vascularity are closely linked to the progression of post-traumatic osteoarthritis and osteonecrosis (93, 96, 97). Changes in PLR homeostasis can alter the subchondral bone and precede changes in joint homeostasis (18–20). When we examined the effect of GC and PTH(1-34) on articular cartilage histologically, no differences in OARSI or Modified Mankin scores were observed. The lack of an effect on articular cartilage may result from biological variables that blunted the effect of GC, as previously mentioned. It is possible that GC and PTH(1-34)-dependent effects on the joint (98–102) would be more apparent with injury, since suppressed PLR exacerbated post-traumatic osteoarthritis in male mice with an osteocyte-intrinsic deletion of transforming growth factor, beta receptor II (*Tgfβr2*) (20).

This study has limitations, including the complexity of biological variables in the effects of GC and PTH(1-34) in the selected conditions, and the need to challenge the joint with injury, age, or diet in order to adequately assess the effect of GC and PTH(1-34) on joint homeostasis. As noted above, some of the effects of GC treatment, including on osteocyte lacunocanalicular outcomes, differed from our prior observations (18) and expectations. Our prior study examined PLR in an established model of GC-induced osteonecrosis (93), whereas the current study employed a less severe GC treatment model to test the ability of PTH(1-34) to recover GC-suppressed PLR. Contrary to the LCN degeneration we previously observed in a model of GC-induced osteonecrosis (18), the effects of GC on the osteocyte LCN were not apparent in the milder conditions chosen here. Though this limits our ability to test the hypothesis that PTH(1-34) mitigates the effects of GC on the LCN, results at the tissue, cellular, and molecular scale consistently show the inability of PTH(1-34) to overcome the effects of GC. Additional studies, such as ptychographic x-ray computed tomography (103), backscatter scanning electron microscopy (12), or confocal imaging of phalloidin/DiI stained bone (104) will be needed to identify strategies to rescue PLR suppression in osteocytes. If identified, PLR agonists may have potential to mitigate the loss of bone and joint homeostasis that occurs with glucocorticoid treatment, aging, or other conditions in which PLR is suppressed.

## Data availability statement

The datasets generated for this study are included in the article/Supplementary Materials. Further inquiries can be directed to the corresponding author. The Nanostring data discussed in this publication have been deposited in the NCBI’s Gene Expression Omnibus (Yee et al., 2025) and are accessible through GEO Series accession number GSE252085 (https://www.ncbi.nlm.nih.gov/geo/query/acc.cgi?acc=GSE252085).

## Ethics statement

The animal study was approved by Institutional Animal Care and Use Committee (IACUC) at the University of California, San Francisco. The study was conducted in accordance with the local legislation and institutional requirements.

## Author contributions

CY: Data curation, Formal analysis, Investigation, Methodology, Visualization, Writing – original draft. CM: Data curation, Formal analysis, Investigation, Writing – review & editing. SK: Data curation, Formal analysis, Investigation, Writing – review & editing. WC: Conceptualization, Data curation, Formal analysis, Funding acquisition, Methodology, Visualization, Writing – review & editing. TA: Conceptualization, Formal analysis, Funding acquisition, Methodology, Visualization, Writing – review & editing.
