# Supplementary material for: The osteocytic actions of glucocorticoids on bone mass, mechanical properties, or perilacunar remodeling outcomes are not rescued by PTH(1-34)
Source: Front Endocrinol (Lausanne). 2024 Jul 18;15:1342938. doi: 10.3389/fendo.2024.1342938 (PMC11291448; doi:10.3389/fendo.2024.1342938)
Supplement: Supplementary file 1 [file DataSheet_1.docx]

Supplementary Material

The osteocytic actions of glucocorticoids on bone mass, mechanical properties, or perilacunar remodeling outcomes are not rescued by PTH(1-34)

Cristal S. Yee *, Christoforos Meliadis, Serra Kaya, Wenhan Chang, Tamara Alliston

*** Correspondence:** Tamara Alliston tamara.alliston@ucsf.edu

**
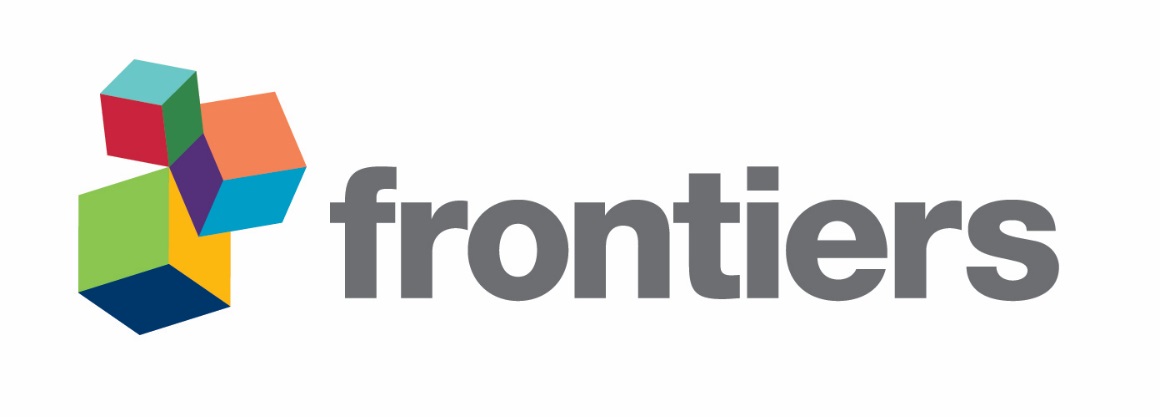
**

# Supplementary Method

**Terminal deoxynucleotidyl transferase dUTP nick end labeling (TUNEL) Stain**

Apoptotic activity was evaluated by terminal deoxynucleotidyl transferase dUTP nick end labeling (TUNEL) stain (Roche cat# 11-684-795-910) on paraffin sections of the right knee as previously described (Fowler et al. 2017; Mazur et al. 2019). Manufacturer’s instructions were followed with permeabilization using 0.1% triton X-100 in 0.1% sodium citrate for 2 mins on ice, and counterstaining with DAPI after staining with TUNEL reaction mixture. As a positive control, samples were treated with recombinant DNase I (1U/ml) in 50mM Tris-HCL, pH7.5, 10mM MgCl2, 1mg/ml BSA for 10 minutes at 25°C to induce DNA strand breaks. Negative controls underwent the TUNEL staining protocol in the absence of terminal transferase. Images were obtained on a Leica DMi8 confocal microscope. Quantification of TUNEL+ cells, normalized to total DAPI+ cells, used three images (40X) from each quadrant of the subchondral bone of the right knee (femur, tibia, medial, lateral). A total of 12 images per animal (n=4 mice/group) were quantified using the ImageJ (RRID: SCR_003070) cell counter plugin. Mean percent TUNEL+ cells over total DAPI+ cells was averaged per quadrant of the knee per animal and averaged within each experimental group.

**Quantitative RT-PCR analysis**

RNA was isolated from bones (humeri from n=7-8 mice/group) as previously described (Dole et al. 2017; Mazur et al. 2019). Briefly, humeri were cleaned free of periosteum, epiphysis were cut off, and marrow removed by centrifugation prior to RNA extraction using the RNeasy mini kit (Qiagen, Valencia, CA) following the manufacturer’s protocol. Nanodrop spectrophotometry was used to obtain equal amounts of RNA to be reverse-transcribed using the iScript cDNA synthesis kit (BioRad). iQ SYBR Green Supermix (BioRad) was used to perform real-time quantitative PCR (qPCR) on a Biorad I-Cycler, with beta-actin as a housekeeping gene used to quantify relative fold change expression of genes of interests (primer sequences in Supplementary Table 1).

**Supplementary Figure 1.**

**Supplemental Figure 1: Nanostring data of gene expression differences in males.**

Volcano plots of Nanostring analysis of mRNA gene expression from humeri of GC **(A)**, PTH(1-34) **(B)**, and GC+PTH(1-34) **(C)** treated male mice (n=4/group) compared to controls. Genes with significant differences in expression level (gray dots) are above the horizontal p-value threshold (dotted gray line) and up-regulated or down-regulated genes fall to either to the right or left sides, respectively, with genes associated with bone remodeling in red dots. **(D)** shows significantly up- and down-regulated gene expression fold changes in each condition. Significant gene expression changes (p-value<0.05) were determined by unpaired t-test between experimental groups, normalized to 7 housekeeping genes (*Gapdh*, *Rpl19*, *Gilz* (*Tsc22d3*), bone sialoprotein (*Ibsp*), beta-2 microglobulin (*B2m*), beta actin (*Actb*), *Serpine2*).

**Supplemental Figure 2: Nanostring data of statistically different gene expression fold changes compared to GC+PTH(1-34) treated females.**

Nanostring analysis highlights significant changes in mRNA levels from humeri of GC **(A)** and PTH(1-34) **(B)** treated female mice (n=4/group) compared to those treated with GC+PTH(1-34). Significant gene expression fold changes (p-value<0.05) were determined by unpaired t-test between experimental groups, normalized to 7 housekeeping genes (*Gapdh*, *Rpl19*, *Gilz* (*Tsc22d3*), bone sialoprotein (*Ibsp*), beta-2 microglobulin (*B2m*), beta actin (*Actb*), *Serpine2*). Individual gene expression changes were obtained by real-time qPCR (n=7-8/group), normalized to beta actin (*Actb*), and presented as mean + SD and two-way ANOVA with post-hoc Holm Sidak **(C-H)**.

**Supplemental Figure 3: TUNEL stain shows no changes in osteocyte apoptosis in GC or PTH(1-34) treated females.**

Negative and positive controls for TUNEL stain show specificity of detecting cellular apoptosis in the subchondral bone plate **(A)**. Representative images of TUNEL stained sections of subchondral bone of the knee in 16-week-old females (n=4/group) show no changes in osteocyte (Ot) (white arrows) or osteoclast (Oc) (white arrowheads) apoptosis across experimental groups **(B,** 40X, scalebar = 100 µm**)**. Quantification (total number of TUNEL positive osteocytes over total number of osteocytes across each quadrant of the knee) is presented as mean + SD and two-way ANOVA with post-hoc Holm Sidak **(C-E)**.

## Supplementary Tables

**Supplemental Table 1. Primer sequences used for gene expression analysis of murine mRNA.**

| **Gene** | **Sequence** |
| --- | --- |
| *Atrogin1* | 5’-AGTGAGGACCGGCTACTGTG-3’  5’-GATCAAACGCTTGCGAATCT-3’ |
| *Dmp1* | 5’-CTGAAGAGAGGACGGGTGATT-3’  5’-CGTGTGGTCACTATTTGCCTG-3’ |
| *Gapdh* | 5'- AGGTCGGTGTGAACGGATTTG -3'  5'- TGTAGACCATGTAGTTGAGGTCA -3' |
| *Mmp13* | 5’-CGGGAATCCTGAAGAAGTCTACA-3’  5’-CTAAGCCAAAGAAAGATTGCATTTC-3’ |
| *Mmp14* | 5’-AGGAGACGGAGGTGATCATCATTG-3’  5’-GTCCCATGGCGTCTGAAGA-3’ |
| *Mmp2* | 5’-AACGGTCGGGAATACAGCAG-3’  5’-GTAAACAAGGCTTCATGGGG-3’ |
| *Murf1* | 5’-CCTGCAGAGTGACCAAGGA-3’  5’-GGCGTAGAGGGTGTCAAACT-3’ |
| *Musa1* | 5’-GAGAAGCCAGGGTTTGAGC-3’  5’-TCATACAGTGTGAGTGCTGCTG-3’ |
| *Tnfsf11 (Rankl)* | 5’-CCAAGATCTCTAACATGACG-3'  5’-CACCATCAGCTGAAGATAGT-3' |
