# Supplementary figures and images for: The osteocytic actions of glucocorticoids on bone mass, mechanical properties, or perilacunar remodeling outcomes are not rescued by PTH(1-34)
Source: Front Endocrinol (Lausanne). 2024 Jul 18;15:1342938. doi: 10.3389/fendo.2024.1342938 (PMC11291448; doi:10.3389/fendo.2024.1342938)

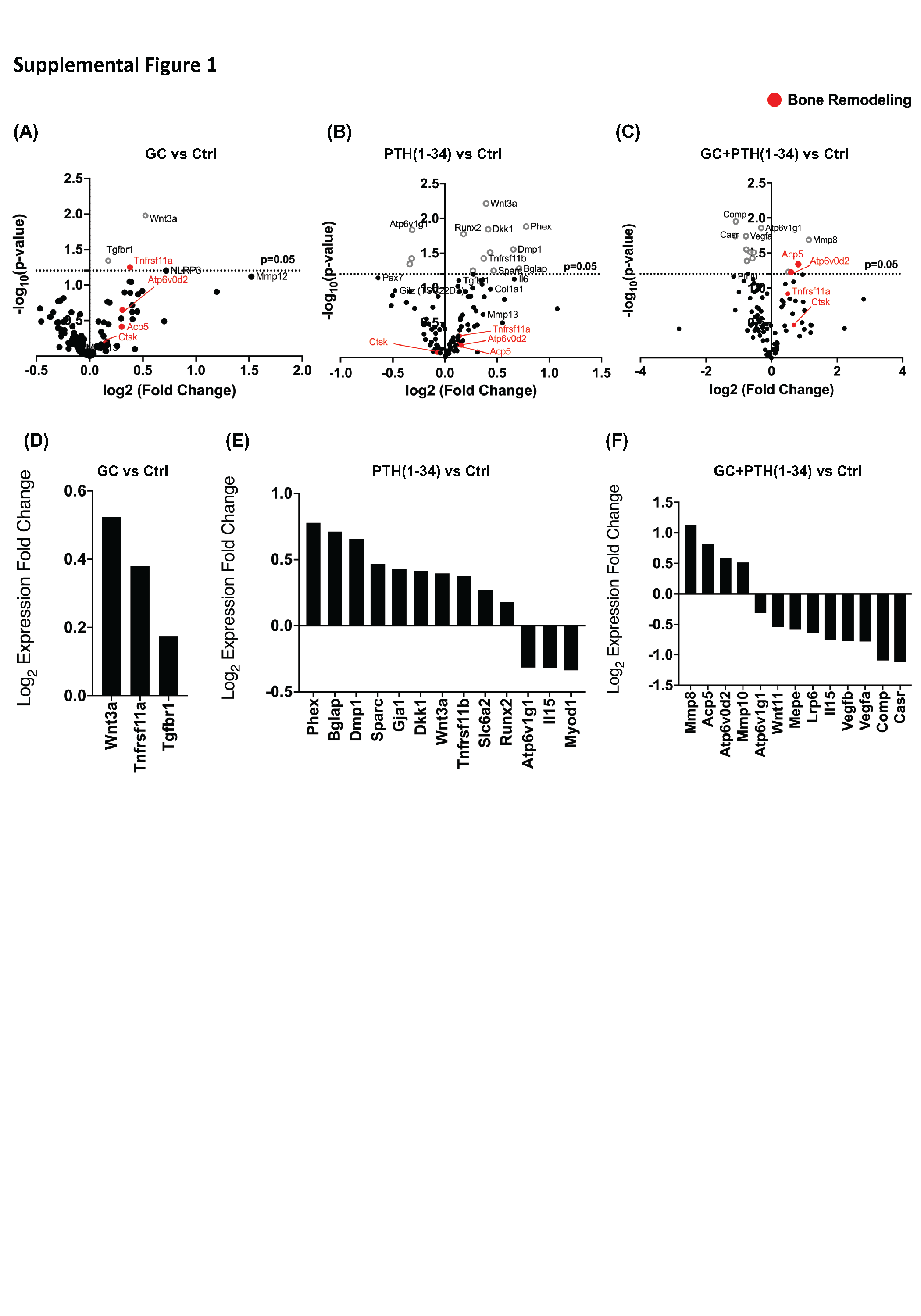

Supplement: Supplementary file 2 [file Image_1.tiff]

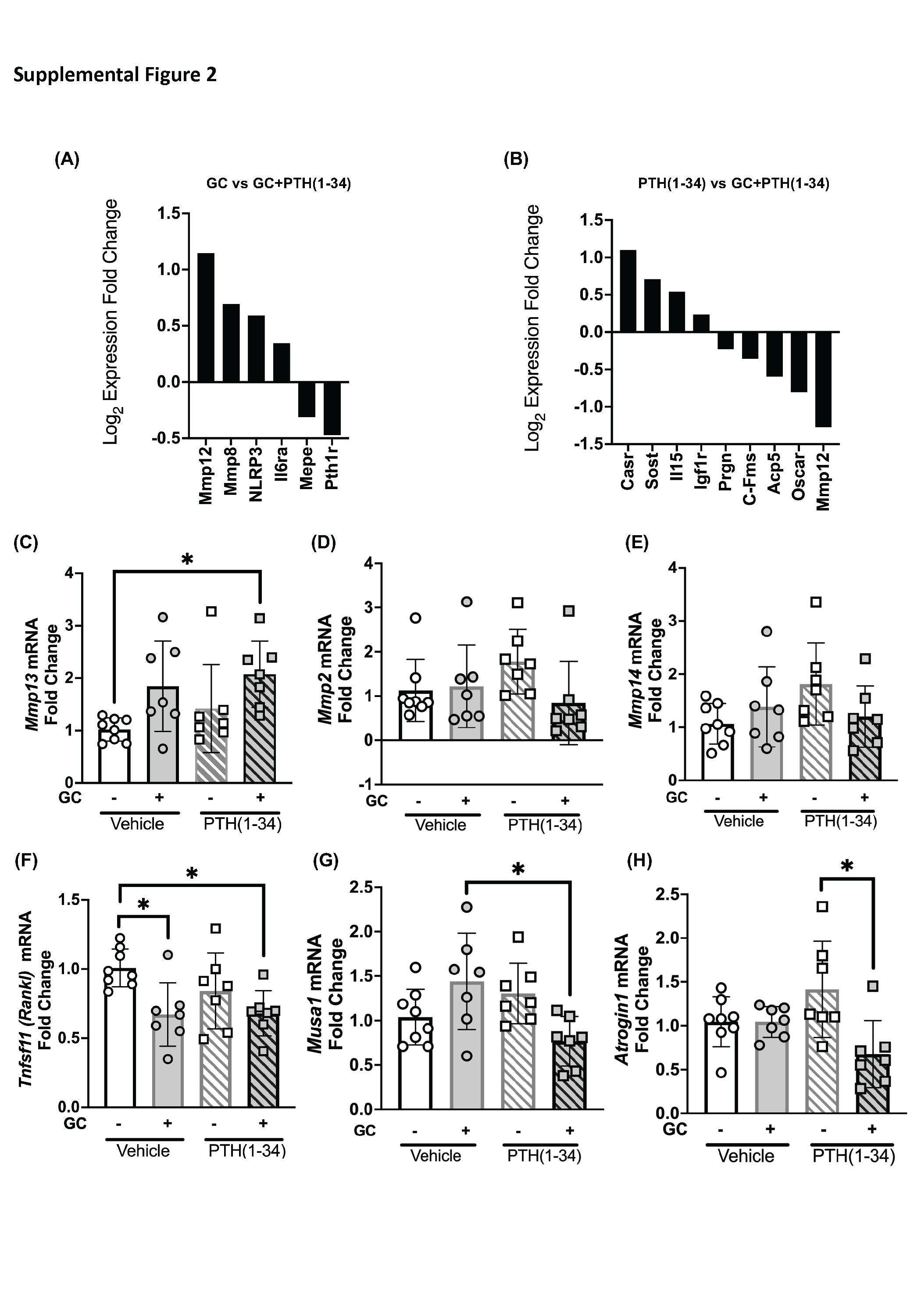

Supplement: Supplementary file 3 [file Image_2.tiff]

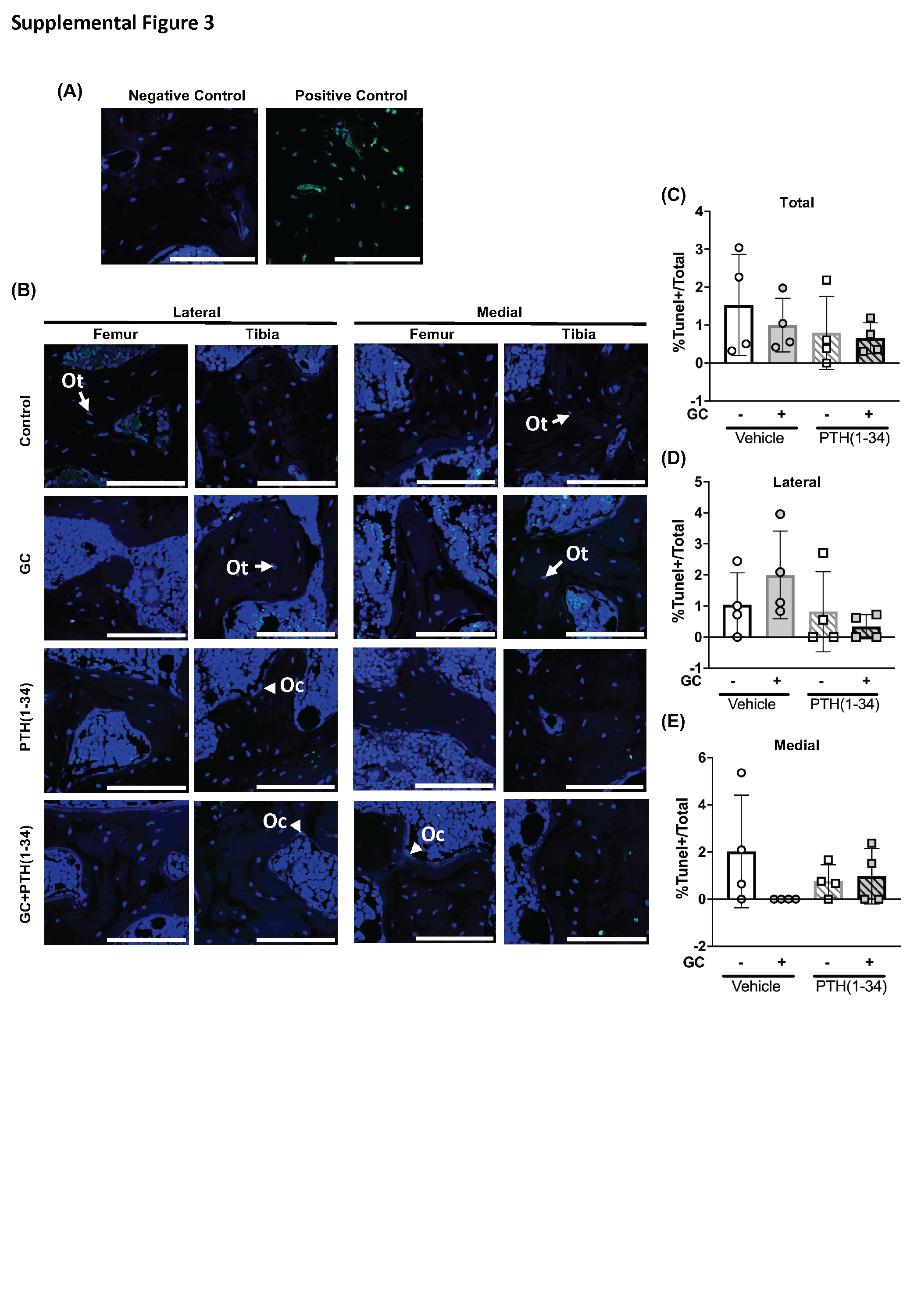

Supplement: Supplementary file 4 [file Image_3.tiff]
